# Percentages of CD4+CD161+ and CD4−CD8−CD161+ T Cells in the Synovial Fluid Are Correlated with Disease Activity in Rheumatoid Arthritis

**DOI:** 10.1155/2015/563713

**Published:** 2015-04-16

**Authors:** Jinlin Miao, Kui Zhang, Feng Qiu, Tingting Li, Minghua Lv, Na Guo, Qing Han, Ping Zhu

**Affiliations:** ^1^Department of Clinical Immunology, Xijing Hospital, Fourth Military Medical University, Xi'an 710032, China; ^2^Department of Neurology, Chinese Navy General Hospital, Beijing 100048, China; ^3^Department of Geriatric Gastroenterology, Chinese People's Liberation Army General Hospital, Beijing 100853, China; ^4^Institute of Basic Medical Science, Xi'an Medical University, Xi'an 710032, China

## Abstract

*Objective*. CD161 has been identified as a marker of human IL-17-producing T cells that are implicated in the pathogenesis of rheumatoid arthritis (RA). This study aimed to investigate the potential link between the percentage of CD161+ T cells and disease activity in RA patients. *Methods*. Peripheral blood (PB) from 54 RA patients and 21 healthy controls was evaluated. Paired synovial fluid (SF) (*n* = 17) was analyzed. CD161 expression levels on CD4+, CD8+, and CD4−CD8− T cells were assessed by flow cytometry. *Results*. The percentage of CD4+CD161+ T cells in RA SF was higher than RA PB, and it was positively correlated with DAS28, erythrocyte sedimentation rate (ESR), and C-reactive protein (CRP). CD4−CD8−CD161+ T cell percentage was decreased in RA PB and was further reduced in RA SF, and its level in SF was inversely correlated with DAS28, ESR, and CRP. However, CD8+CD161+ T cell percentage was neither changed in RA PB and SF nor correlated with disease activity indices. *Conclusion*. An increased CD4+CD161+ T cell percentage and a decreased CD4−CD8−CD161+ T cell percentage are present in RA SF and are associated with disease activity, and the accumulation of CD4+CD161+ T cells in SF may contribute to the local inflammation of RA.

## 1. Introduction

Rheumatoid arthritis (RA) is a systemic inflammatory disease characterized by joint inflammation of synovial tissue eventually leading to joint damage and functional disability. Multiple innate and adaptive effector cells, including macrophages, neutrophils, fibroblasts, B cells, and T cells, play important roles in the pathogenesis of RA [1]. T helper-type 17 (Th17) cells, a distinct subset of Th cells producing interleukin- (IL-) 17 in humans, may be involved in the pathogenesis of autoimmune and chronic inflammatory disorders, including RA [2, 3]. Numerous clinical studies including our data have demonstrated that the percentage of Th17 cells in RA patients was elevated and positively correlated with the degree of local and systemic disease activity [4–6]. Moreover, IL-17, the characteristic cytokine of Th17 cells, was implicated in the pathogenesis of RA [7]. IL-17A is a proinflammatory cytokine expressed in synovial membrane cultures of RA patients [8] and synovial tissue IL-17 is associated with more rapid joint damage progression in synergy with tumor necrosis factor- (TNF-) *α* [9]. An enhanced expression of IL-17 has also been observed in the synovial fluid of RA patients [8, 10], and IL-17 has become a new therapeutic target for mouse RA models and human RA [11].

CD161 is the human equivalent of mouse NK cell receptor P1A and constitutes a type II transmembrane glycoprotein with characteristics of the C-type lectin superfamily [12]. CD161 was one of the most upregulated genes in human Th17 cells compared to Th1 or Th2 cells and its expression is induced by RAR-related orphan receptor C (RORC), the Th17 lineage transcription factor [13, 14]. In addition, human Th17 cells exclusively originate from CD4+CD161+ naive T cell progenitors, and CD161 is a novel surface marker for Th17 cells [13, 14]. Moreover, Maggi et al. provided evidence that CD161 is a marker of all human IL-17-producing T cell subsets, including CD3+CD4+CD8−, CD3+CD4−CD8+, and CD3+CD4−CD8− cells [14]. It has been reported that circulating CD4+CD161+ T cells are increased in seropositive arthralgia patients but decreased in newly diagnosed RA patients [15]. Furthermore, this study showed that CD4+CD161+ T cells were enriched in synovial fluid (SF), while CD8+CD161+ T cells were not accumulated in SF of RA patients [15]. In fact, we have previously demonstrated that RA patients seemed to have higher percentages of circulating CD161+ cells in CD4+ T cells than healthy controls, but the difference did not reach statistical significance [16]. However, little was known about the percentages of CD161 expressing T cell subsets (including CD3+CD4+, CD3+CD8+, and CD3+CD4−CD8− cells) in blood and the local site of inflammation of RA patients and their potential link to disease activity.

Therefore, we explored the percentages of CD161 expressing T cell subsets in PB and SF of RA patients and assessed their correlations with the degree of disease activity.

## 2. Materials and Methods

### 2.1. Patients

Samples of peripheral blood (PB) were obtained from 54 RA patients and from 21 age- and sex-matched healthy controls. And synovial fluid (SF) samples were obtained from the knee joints of 17 patients with active RA. All patients fulfilled the 1987 revised criteria of the American College of Rheumatology [17]. Disease activity was assessed by the 28-joint disease activity score (DAS28) on the day of sample collection. Erythrocyte sedimentation rate (ESR) and C-reactive protein (CRP) were determined on the day of sample collection in the clinical laboratory. The study conforms to the recommendations of the Declaration of Helsinki. The Ethics Committee of Xijing Hospital approved this study, and the informed consent from all subjects was obtained.

### 2.2. Preparation of Mononuclear Cells

SF samples were treated with 20 *µ*g/mL hyaluronidase (Sigma-Aldrich, St. Louis, MO, USA) for 30 min at 37°C, and cells were then washed twice with phosphate-buffered saline. SF mononuclear cells (SFMCs) and PB mononuclear cells (PBMCs) were isolated from sodium heparinized whole blood and SF cell suspension samples using Ficoll-Paque density gradient centrifugation (GE Healthcare, Pittsburgh, PA, USA) by standard procedures.

### 2.3. Flow Cytometric Analysis of T Cell Surface Markers

The phenotypes of lymphocytes in PB and SF were determined using flow cytometry. Briefly, PBMCs and SFMCs were stained with the following fluorochrome conjugated monoclonal antibodies: fluorescein isothiocyanate- (FITC-) conjugated CD3 (SK7), peridinin chlorophyll protein- (PerCP-) conjugated CD4 (SK3), allophycocyanin- (APC-) conjugated CD8 (SK1), phycoerythrin- (PE-) conjugated CD161 (DX12), and isotype-matched control IgG antibodies (all from BD Biosciences, San Diego, CA, USA) for 30 min at room temperature, according to the manufacturer's instructions. Stained cells were analyzed using FACSCalibur flow cytometer (BD Biosciences), and data analysis was performed with Cell Quest software (BD Biosciences).

### 2.4. Statistical Analysis

Differences between groups were determined using the nonparametric Mann-Whitney test. Paired samples were compared using a Wilcoxon matched pairs signed rank sum test. Correlations were evaluated by nonparametric Spearman's correlation analysis. Data analyses were performed using GraphPad Prism version 5.0 (GraphPad Software, San Diego, CA, USA). For all tests, a two-sided* P* value less than 0.05 was considered significant.

## 3. Results

### 3.1. Subject Basic Characteristics

Clinical characteristics of RA patients and healthy controls are illustrated in Table 1. Fifty-four patients with RA and 21 healthy controls (HC) were recruited, and synovial fluid (SF) samples were obtained from 17 active RA patients. There was no significant difference in age and gender between the three groups. In addition, disease duration, positive rate of RF and anti-CCP antibodies, and proportion of patients previously using medications were comparable between the group of total RA patients and the group of those patients with collected SF. In addition, ESR levels tended to be increased in RA patients with collected SF (*P* = 0.052), and CRP and DAS28 levels were significantly higher in RA patients with collected SF than in total RA patients (*P* = 0.032 and* P* = 0.017, resp.).

### 3.2. Percentage of Circulating CD161+ T Cells in RA Patients and Healthy Controls

**Figure 1:**
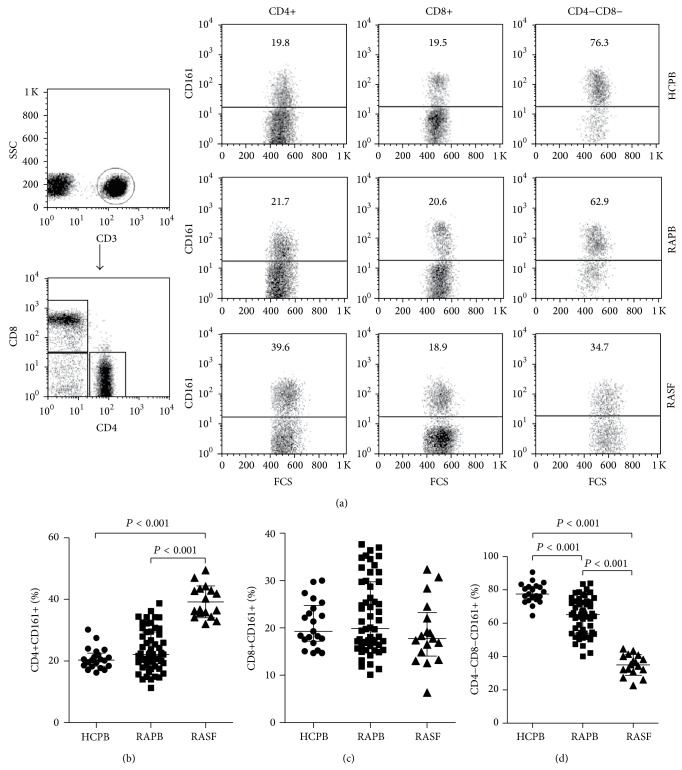
Presence of CD161+ T cell subsets in RA patients and HC. (a) Flow cytometric dot-plots show gating strategy: CD3+ T cells were gated using side scatter profile and the expression of CD3; then CD4+, CD8+, and CD4−CD8− T cells were gated based on their expression of CD4 and CD8, and the CD161 expression levels in these T cell subsets were analyzed from representative HC peripheral blood (PB), RA PB, and RA synovial fluid (SF). Percentages of CD4+CD161+ (b), CD8+CD161+ (c), and CD4−CD8−CD161+ T cells (d) in HC PB, RA PB, and RA SF. Horizontal line indicates median value.* P* values were assessed by Mann-Whitney* U* test.

First, we assessed circulating CD3+ T cell subsets expressing the IL-17 producing cells marker CD161 in RA patients and HC, and representative examples of flow cytometric dot-plots are shown in Figure 1(a). The percentage of circulating CD4+CD161+ (22.19, 18.41–29.44%) (median, interquartile range) and CD8+CD161+ cells (19.90, 16.26–29.74%) in RA patients was not different from HC (20.34, 18.35–22.58%, and 19.27, 17.19–24.27%;* P* = 0.122 and* P* = 0.675, resp.) (Figures 1(b) and 1(c)), while the percentage of CD4−CD8−CD161+ cells was significantly lower in RA patients (65.22, 53.92–72.81%) than in HC (77.54, 73.92–82.14%;* P* < 0.001) (Figure 1(d)).

### 3.3. Percentage of CD161+ T Cells at the Site of Inflammation in RA

**Figure 2:**
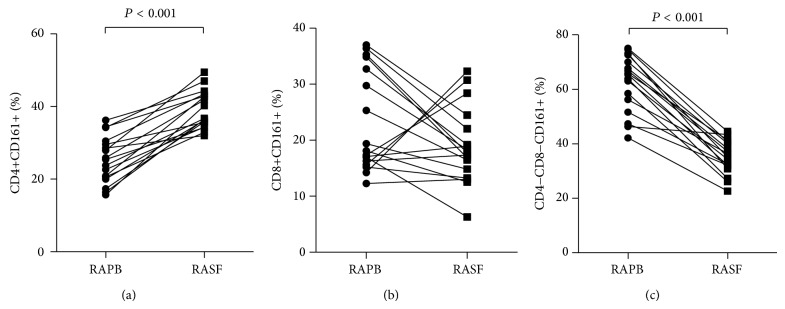
The percentages of CD4+CD161+ (a), CD8+CD161+ (b), and CD4−CD8−CD161+ T cells (c) in paired RA PB and SF samples are shown.* P* values were assessed by Wilcoxon matched pairs signed rank sum test.

CD161 may function as an adhesion molecule and is involved in transendothelial migration [18, 19]. Then, the relative percentages of CD161 expression T cells in SF from patients with RA were assessed. The percentage of CD4+CD161+ cells in the RA SF was significantly increased (36.71, 34.99–43.18%) as compared to HC PB (*P* < 0.001), total RA PB (*P* < 0.001), and paired RA PB (25.43, 20.33–30.04%;* P* < 0.001) (Figures 1(b) and 2(a)), while a significantly lower percentage of CD4−CD8−CD161+ cells was observed in the RA SF (35.50, 31.49–40.45%) than in HC PB (*P* < 0.001), total RA PB (*P* < 0.001), and paired PB (65.41, 53.91–71.36%;* P* < 0.001) (Figures 1(d) and 2(c)). However, there were no significant differences in the percentage of CD8+CD161+ T cells among HC PB, total RA PB, RA SF (17.75, 14.06–23.27%) and paired PB (18.12, 15.96–33.80%) (all *P* > 0.05) (Figures 1(c) and 2(b)).

### 3.4. Correlations of CD161+ T Cells with Disease Activity in RA Patients

Then, we assessed if the presence of CD161+ T cells in SF was correlated with systemic markers of disease activity (Table 2). The percentage of CD4+CD161+ cells in SF was positively correlated with DAS28 (*r* = 0.689,* P* = 0.002), ESR (*r* = 0.569,* P* = 0.017), and CRP levels (*r* = 0.679,* P* = 0.003). In contrast, the percentage of CD4−CD8−CD161+ cells in SF was correlated inversely with DAS28 (*r* = −0.671,* P* = 0.003), ESR (*r* = −0.632,* P* = 0.007), and CRP levels (*r* = −0.663,* P* = 0.004). However, no correlations were present between percentages of CD8+CD161+ cells in SF and DAS28 (*r* = 0.137,* P* = 0.599), ESR (*r* = −0.199,* P* = 0.445), and CRP levels (*r* = 0.074,* P* = 0.779). Additionally, there were no correlations between systemic markers of disease activity and CD161+ T cells in PB (all* P* > 0.05) (Table 2).

## 4. Discussion

Ample previous studies have indicated that Th17 cells and IL-17 critically contribute to the pathogenesis of RA [2–11]. Furthermore, not only CD4+ cells but also CD8+ and CD4−CD8− T cells that produce IL-17 express the CD161 on their surface, CD161 thus is considered a marker of all IL-17-producing T cells [14]. Moreover, it has been reported that the expression of CD161 is maintained in the life cycle of human IL-17-producing T cells [13, 14, 18]. Therefore, CD161 expression may represent a way to detect IL-17-producing T cell ancestry in circulating and tissue infiltrating T cells.

We hypothesized that CD161 expressing T cells representing IL-17-producing T cells act on the pathogenesis of RA, and the levels of CD161 expression T cells in circulation or at inflammatory sites may be regulated prior to or after the development of inflammatory arthritis. Therefore, we assessed the percentage of circulating CD161 expression T cells in HC and RA patients first. Our results showed that the percentage of circulating CD4+CD161+ cells was not different from HC, as we previously reported [16]. However, conflicting data have been reported in a recent study which showed that patients with newly diagnosed RA had decreased levels of CD4+CD161+ cells [20]. Some of this variation may be due to the study cohort. The previous study recruited patients with newly diagnosed RA (with a mean duration of preceding symptoms of 10.2 months), while our cohort had a median disease duration of 66 months and thus may be more consistent with patients with established disease. In addition, this variation may be explained by treatment effects, as most of patients in our study were treated with DMARDs (Table 1), while the patients in the previous study were treatment-naïve [20].

CD161 may function as an adhesion molecule and thereby facilitates extravasation and tissue localization [18, 19]. And a recent study reported that CD161 is a receptor expressed on different T cell subsets and may be involved in the pathogenesis of a given disease [21]. Hence, we investigated the performance of CD161 expressing T cells from the synovial fluid of RA patients as representative cells from the RA inflammatory site. Indeed, synovial fluid from active RA patients was found to be enriched in CD4+CD161+ T cells, which is in agreement with the data of a recent study [20]. Moreover, the percentage of CD4+CD161+ cells was positively correlated with DAS28, ESR, and CRP levels in SF of RA patients. These findings suggest that extravasation and migration of CD4+CD161+ T cells to the joints may be facilitated by CD161 mediated adhesion [19] and indicate that CD4+CD161+ T cells, as Th17 precursor cells, may play a pathogenic role at the local site of inflammation in RA. Moreover, the proportions of CD4+CD161+ T cells in SF might reflect the degree of disease activity in RA patients. Thus, we believe that further studies are needed to assess whether the CD4+CD161+ population was associated with joint damage progression, such as cartilage damage and bone erosion.

Meanwhile, levels of CD8+CD161+ cells and CD4−CD8−CD161+ cells in PB and SF were also examined in this study. Similar to a previous study [22], our data showed that the percentage of circulating CD8+CD161+ T cells in RA patients was not different from HC. In addition, no differences were observed between RA SF and paired RA PB or total RA PB of CD8+CD161+ T cells. Furthermore, there was no correlation between CD8+CD161+ T cell percentage and disease activity in SF or PB of RA. This could be due to the relatively limited number of patients, but an intrinsic phenomenon is possible as well.

As reported in the literature, upon activation of the T cell receptor, a small number of CD3+ T cells that are CD4 and CD8 double negative have the capacity to produce IL-17 [23]. Furthermore, IL-17-producing cells were only found in the CD161+ cell fraction of CD4−CD8− cells, and CD4−CD8− T cells have the highest mRNA expression of RORC and IL-23R when compared to CD4+ and CD8+ T cells [14]. In addition, IL-17-producing CD4−CD8− T cells were expanded and involved in the pathogenesis of kidney damage and salivary gland damage in patients with systemic lupus erythematosus and Sjögren's syndrome [24, 25]. However, we demonstrated that the percentage of circulating CD4−CD8−CD161+ cells, representing IL-17-producing CD4−CD8− T cell ancestry cells, was significantly decreased in RA patients when compared to HC. To our surprise, there was no correlation between circulating CD4−CD8−CD161+ cell percentage and the disease activity indices of RA. Furthermore, CD4−CD8−CD161+ cell percentage in RA SF was further decreased as compared to paired RA PB and total RA PB, and this reduction in SF was negatively correlated with DAS28, ESR, and CRP levels in RA patients. These findings suggest that the pathogenesis of RA may differ from that of other rheumatic diseases, and further investigations are needed to determine the mechanism for decreased CD4−CD8−CD161+ cell percentage and the performance of IL-17-producing CD4−CD8− T cells in RA patients. In addition, these results indicate that CD4+CD161+ and CD4−CD8−CD161+ T cells in the joint fluid may be considered as local parameters of joint inflammation, whereas CRP and ESR levels were regarded as systemic parameters of inflammation. More importantly, these data point towards a potentially important role for CD4+CD161+ and CD4−CD8−CD161+ T cells as regulators of joint inflammation and RA pathogenesis.

## 5. Conclusion

In conclusion, our results demonstrated that an increased percentage of CD4+CD161+ T cells and a decreased percentage of CD4−CD8−CD161+ T cells are present in SF of RA patients and correlate well with disease activity indices, and thus, may be involved in the local inflammation and clinical outcome of RA. These data suggest that CD4+CD161+ and CD4−CD8−CD161+ T cell levels in SF may reflect the degree of disease activity and joint inflammation in RA patients. Further studies are required to clarify the pathogenetic role of CD4+CD161+ and CD4−CD8−CD161+ T cells and investigate the mechanism for their change in RA.

## Figures and Tables

**Table 1:** Characteristics of rheumatoid arthritis (RA) patients and healthy controls (HC).

Characteristics	HC	RA	SF RA
Number of patients	21	54	17
Age in years, median (IQR)	45.0 (34.0–53.0)	46.5 (36.5–54.8)	47.0 (42.0–54.5)
Female sex, *n* (%)	15 (71.4)	40 (74.1)	13 (76.5)
Disease duration, mo, median (IQR)	na	66.0 (11.8–115.5)	60.0 (19.0–102.0)
Rheumatoid factor positive, *n* (%)	na	38 (70.4%)	12 (70.6%)
Anti-CCP positive, *n* (%)	na	40 (74.1%)	14 (82.4%)
ESR, mm/hour, median (IQR)	na	26.5 (14.8–54.0)	54.0 (25.5–72.0)
CRP, mg/dL, median (IQR)	na	0.6 (0.3–3.6)	3.1 (0.5–5.7)^∗^
DAS28, median (IQR)	na	4.5 (2.6–5.7)	5.4 (4.5–6.1)^∗^
Systemic steroids, *n* (%)	na	7 (13.0)	2 (11.8)
NSAIDs, *n* (%)	na	8 (14.8)	3 (17.6)
DMARDs (excluding anti-TNF), *n* (%)	na	43 (79.6)	13 (76.5)
Anti-TNF-*α* therapy, *n* (%)	na	8 (14.8)	3 (17.6)

Values are presented as median (interquartile range) or number (percentage). SF, synovial fluid; IQR, interquartile range; Anti-CCP, anticyclic citrullinated peptide antibodies; ESR, erythrocyte sedimentation rate; CRP, C-reactive protein; DAS28, 28-joint disease activity score; NSAIDs, nonsteroidal anti-inflammatory drugs; DMARDs, disease-modifying antirheumatic drugs; TNF-*α*, tumor necrosis factor-*α*; na, not applicable. ^∗^ *P* < 0.05 compared to RA patients.

**Table 2:** Correlations between percentages of CD161+ T cell subsets in RA synovial fluid (SF) and peripheral blood (PB) and DAS28, ESR, and CRP.

	DAS28		ESR		CRP	
	*r*	*P*	*r*	*P*	*r*	*P*
SF						
CD4+CD161+	0.689	**0.002**	0.569	**0.017**	0.679	**0.003**
CD8+CD161+	0.137	0.599	−0.199	0.445	−0.074	0.779
CD4−CD8−CD161+	−0.671	**0.003**	−0.632	**0.007**	−0.663	**0.004**
PB						
CD4+CD161+	0.224	0.103	0.099	0.477	0.137	0.324
CD8+CD161+	0.106	0.445	0.044	0.752	0.167	0.227
CD4−CD8−CD161+	−0.191	0.166	−0.104	0.453	−0.125	0.367
